# Circular CDC like kinase 1 suppresses cell apoptosis through miR-18b-5p/Y-box protein 2 axis in oral squamous cell carcinoma

**DOI:** 10.1080/21655979.2022.2027174

**Published:** 2022-02-13

**Authors:** Lixin Guo, Qing Lin, Xiqun Zhao, Jing Xu

**Affiliations:** a Scientific Education Section, Jinan stomatological hospital; b Department of Endodontics, Jinan stomatological hospital Gao Xin branch; c Pediatric Dentistry, Jinan Stomatological Hospital; d Department of stomatology, Shengli olifield central hospital

**Keywords:** Circ-CLK1, oral squamous cell carcinoma, apoptosis

## Abstract

This study aimed to explore the role of circular-CDC like kinase 1 (circ-CLK1) in the pathogenesis of oral squamous cell carcinoma (OSCC). Circ-CLK1 expression levels were detected via reverse transcription-quantitative polymerase chain reaction (RT-qPCR). The effects of circ-CLK1 knockdown on the viability and apoptosis of OSCC cells were determined using the cell counting kit-8 (CCK-8) assay, EdU staining, flow cytometry, and Western blotting. StarBase and TargetScan were used to predict targeting relationships, which were then confirmed by the dual luciferase reporter assay and RNA pull-down assay. We found that the expression of circ-CLK1 was significantly higher in OSCC patients and cell lines. Inhibition of circ-CLK1 reduced the viability and proliferation of OSCC cells while enhancing their apoptosis. However, inhibiting miR-18b-5p or overexpression of Y-box protein 2 (YBX2) can reverse the effect of circ-CLK1 knockdown on OSCC cells. Therefore, circ-CLK1 inhibited the apoptosis of OSCC cells through the miR-18b-5p/YBX2 axis, and these findings suggest that circ-CLK1 could be a potential therapeutic target for OSCC patients.

## Introduction

Oral squamous cell carcinoma (OSCC) is the most common oral malignant tumor that usually originates from the mucosa and occurs on the tongue and in the oral cavity [1,2]. Smoking and drinking are the main risk factors for OSCC [3]. There are approximately 300,000 new cases of oral cancer each year, of which OSCC accounts for more than 80% [4,5]. OSCC usually occurs in people over 50 years of age, but in recent years, patients tend to be younger [6,7]. Currently, the main clinical treatment for OSCC is surgery, preoperative or postoperative adjuvant radiotherapy, and chemotherapy [8]. However, OSCC is highly invasive. Most patients are already in the late stage of clinical treatment when diagnosed, so the 5-year survival rate is low [9]. Therefore, the treatment of OSCC requires further investigation.

Apoptosis is an active programmed cell death controlled by genes [10], mainly manifested as the activation of the caspase family, DNA damage, protein crosslinking, and mitochondrial membrane potential change [11]. Apoptosis mainly involves exogenous and endogenous pathways mediated by death receptors and mitochondria, respectively [12]. Apoptosis disorders in the human body are often related to the occurrence and development of tumors [13], and in OSCC patients, apoptosis is also significantly increased [14]. Therefore, research on apoptosis is important for OSCC treatment.

Circular RNA (circRNA) is a non-coding RNA with a circular structure that is present in eukaryotic cells of various organisms and is distributed more in the cytoplasm than in the nucleus [15]. CircRNAs are usually composed of 1–5 exons or 1–2 introns and contain intergenic or non-coding components [16,17]. Due to their unique structure, circRNAs are more stable than linear RNAs [18] and are highly conserved in evolution [19]. It was found that circRNA acts as a sponge to competitively bind microRNA (miRNA), thereby affecting the role of miRNAs on target genes [20]. It has been reported that circRNAs are involved in the regulation of various tumors [21,22]. In OSCC, a variety of circRNAs have been reported to participate in the pathogenesis of tumors through competing RNA (ceRNA) mechanisms [23,24]. Circular-CDC like kinase 1 (circ-CLK1) is a circRNA located on chromosome 2, which has not been reported in OSCC.

Therefore, this study aimed to explore the molecular mechanism by which circ-CLK1 regulates OSCC development as well as its role in apoptosis. We hypothesized that circ-CLK1 inhibited the apoptosis of OSCC cells through the miR-18b-5p/YBX2 axis.

## Materials and methods

### Patients

Clinical samples were collected from OSCC patients (n = 48) and healthy volunteers (n = 48) at Shengli Oilfield Central Hospital. Additionally, the clinical information of OSCC patients were collected. The study was approved by the Ethics Committee of the Shengli Oilfield Central Hospital. Signed informed consent was obtained from each participant.

### Cell culture

OSCC cell lines (UM1 and HSC-2) and normal human oral epithelial cell lines (SG) were purchased from the Type Culture Collection of the Chinese Academy of Sciences (Shanghai, China). All cells were cultured in DMEM (Thermo Fisher Scientific, USA). FBS (10%) (Gibco, Waltham, MA, USA) and 1% penicillin/streptomycin (Gibco, Waltham, MA, USA) were added to the medium. Cells were maintained in 5% CO_2_ at 37°C.

### Reverse transcription-quantitative polymerase chain reaction (RT-qPCR)

RT-qPCR was performed according to a previous study [25]. RNA samples were extracted from all the cells using a commercially available kit (Takara, Japan). Then, cDNA was synthesized and PCR was performed using a Real-Time PCR Detection System (Bio-Rad, USA). The primer sequences used were as follows..

circ-CLK1: F: 5′-TGAGGAGGGTCACCTGATCT-3′, R: 5′-ATTTCCAAATCCCTGAAAGCTTAAT-3′;

miR-18b-5p: F: 5′-GCGTAAGGTGCATCTAGTGCAG-3′, R: 5′-GTCGTATCCAGTGCAGGGTCCGAG-3′;

YBX2: F: 5′-AGAGGTGGCAGCAAAAGAAA-3′, R: 5′- GTGCCCTCTATAGGCTGCTG-3′;

GAPDH: F: 5′-GAGTCCACTGGCGTCTTCAC-3′, R: 5′-ATCTTGAGGCTGTTGTCATACTTCT-3′

### Cell viability

According to previous study [26], OSCC cells were resuspended and seeded in 96-well plates at 100 μl/well. Ten microliters of CCK8 reagent (AmyJet Technology Co., Ltd.) was added to each well and cultured for 4 h at 37°C. A microplate reader (Nanjing DeTie Experimental Equipment Co., Ltd.) was used to measure absorbance at 450 nm.

### Cell death

OSCC cells were stained using the PI/ Annexin V-FITC kit (Sigma) in the dark for 10 min at room temperature [27]. After that, the cells were counted using flow cytometry (BD Biosciences).

### Flow cytometry assay

The TransDetect® Annexin V-FITC/PI Kit (FA101-01; TransGen Biotech Co., Ltd.) was used to measure the apoptosis of UM1 and HSC-2 cells according to a previous study [28]. Briefly, 5 μL Annexin V-FITC was added to a 6-well plate, and the cells were cultured for 15 min in the dark at room temperature. The apoptosis rates of OSCC cells were determined using a NovoCyte Advanteon B4 Flow Cytometer and NovoSampler Q software (Agilent Technologies Co., Ltd.).

### Western blot

According to a previous study [29]. Protein extracts were subjected to 10% SDS gel electrophoresis. The protein extracts were then transferred to a polyvinylidene fluoride membrane (Millipore) and incubated with primary antibodies at 4°C overnight. The following day, the membrane was incubated with the secondary antibodies for 2 h at room temperature. Finally, the images were captured using an ECL system (Thermo Fisher Scientific, Inc.).

### Dual luciferase reporter assay

The wild-type (WT) and mutant (MUT) type 3-UTR regions of circ-CLK1 and Y-box protein 2 (YBX2) luciferase reporter vectors were designed and synthesized by Guangzhou RiboBio Co., Ltd. After cultivation for 24 h, the cells were lysed. The Luciferase Reporter Assay Kit (K801-200; BioVision Tech Co., Ltd.) was used to analyze luciferase activity 48 h after co-transfection with miR-18b-5p mimic/control and the luciferase reporter vectors. Finally, luciferase activity was normalized to Renilla luciferase activity [30].

### RNA pull-down

The MagCapture RNA Pull Down Assay Kit (297–77501; Whatman Co., Ltd.) was used for the RNA pull-down assay according to the manufacturer’s protocols [31]. Proteins were then collected for mass spectrometry analysis.

### Statistical analysis

Each experiment was carried out 3 times. All data were calculated using GraphPad Prism and are presented as mean ± SD. The Student’s t-test was used to compare the differences between the two groups, and the analysis of variance (ANOVA) followed by Duncan’s post-hoc test was used for multiple groups. Significance was set at *P* < 0.05.

## Results

This study demonstrated that circ-CLK1 can serve as a potential biomarker for OSCC, and the mechanism by which circ-CLK1 regulates apoptosis through the miR-18b-5p/YBX2 axis promotes the development of OSCC, which is important for molecular targeted therapy of OSCC.

### Circ-CLK1 was highly expressed in OSCC

**Figure 1.**
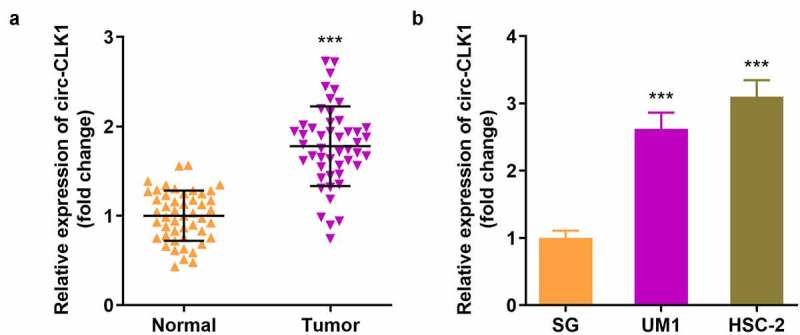
**Circular-CDC like kinase 1 (circ-CLK1) is highly expressed in oral squamous cell carcinoma (OSCC)**. (a) circ-CLK1 expression in OSCC patients; (b) circ-CLK1 expression in UM1 and HSC-2 cells. *** *P* < 0.001 versus control.

We collected 48 samples from patients and healthy volunteers, and found that the expression of circ-CLK1 was markedly increased in OSCC patients (Figure 1a). In addition, circ-CLK1 expression was notably increased in UM1 and HSC-2 cells (Figure 1b). Besides, as shown in Table 1, we found high levels of circ-CLK1 were significantly assoiated with Lymph node metastasis and TNM stage.

**Table 1.** Correlation between circ-CLK1 expression and clinicopathological characteristics of OSCC patients

**Clinicopathologic characteristics**	**n**	circ-CLK1		
		**Low(n = 18)**	**High(n = 30)**	**p-value**
Age (years)				0.8811
< 60	22	8	14	
≥ 60	26	10	16	
Sex				0.3643
Male	20	6	14	
Female	28	12	16	
Smoking				0.9393
Nonsmoker	29	11	18	
Smoker	19	7	12	
Tumor site				0.3914
Tongue	17	5	12	
Nontongue	31	13	18	
Drinking				0.2016
nondrinker	21	10	11	
Drinker	27	8	19	
Lymph node metastasis				0.0343*
Yes	28	7	21	
No	20	11	9	
TNM stage				0.0305*
I–II	25	13	12	
III–IV	23	5	18	
Tumor grade				0.1387
Well/moderate	31	14	17	
Poor	17	4	13

### Knockdown of circ-CLK1 reduced tumor cell viability and proliferation and increased apoptosis

**Figure 2.**
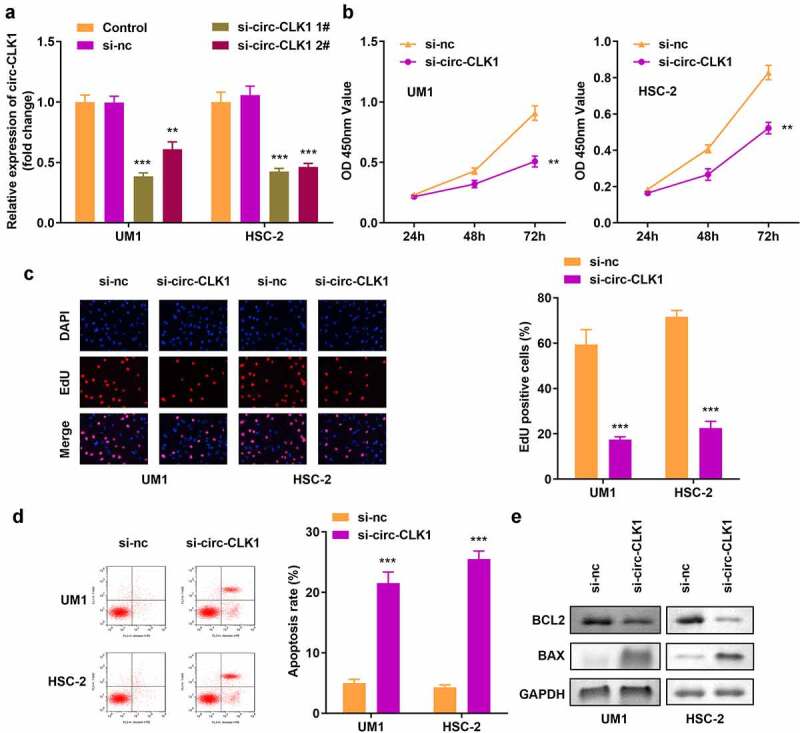
**Knockdown of circular-CDC like kinase 1 (circ-CLK1) reduces tumor cell viability, proliferation while increased apoptosis**. (a) Expression of circ-CLK1 in UM1 and HSC-2 cells; (b) Cell viability of UM1 and HSC-2 cells; (c) Cell proliferation of UM1 and HSC-2 cells; (d) Apoptosis of UM1 and HSC-2 cells; (e) Protein expression of Bcl-2-associated X protein (BAX) and B-cell lymphoma-2 (BCL2). **P < 0.01, *** *P* < 0.001 versus control.

The expression of circ-CLK1 was notably decreased compared with the blank vector, indicating the success of transfection, and 1# was used in the following experiments (Figure 2a). Knockdown of circ-CLK1 markedly reduced the viability and proliferation of UM1 and HSC-2 cells (Figure 2b, 2c). Figure 2d shows that more apoptosis occurred in tumor cells, B-cell lymphoma-2 (BCL2) protein expression was decreased, and Bcl-2-associated X protein (BAX) expression was increased (Figure 2e).

### Circ-CLK1 directly targeted miR-18b-5p

**Figure 3.**
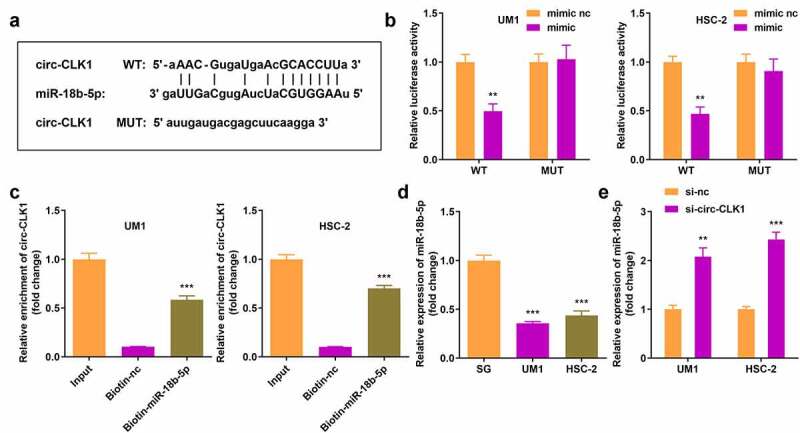
**Circular-CDC like kinase 1 (circ-CLK1) directly targets miR-18b-5p**. (a) Binding sites of circ-CLK1 and miR-18b-5p; (b) The luciferase activity of UM1 and HSC-2 cells co-transfected with luciferase reporter vector containing circ-CLK1 miRNA response elements (MREs) for miR-18b-5p and miR-18b-5p overexpression vector; (c) Circ-CLK1 expression after pulling down with miR-18b-5p; (d) miR-18b-5p expression in UM1 and HSC-2 cells. (e) miR-18b-5p expression in circ-CLK1 knockdown cells. **P < 0.01, *** *P* < 0.001 versus control.

We predicted the target miRNAs of circ-CLK1, and Starbase 3.0 revealed that circ-CLK1 possessed various miRNA response elements (MREs) for miR-18b-5p (Figure 3a). Then, dual luciferase reporter and RNA pull-down assays were performed for further validation (Figure 3b, 3c). In addition, miR-18b-5p expression was significantly decreased in UM1 and HSC-2 cells (Figure 3d), while it was increased in circ-CLK1 knockdown tumor cells (Figure 3e).

### miR-18b-5p inhibition reversed the effects of circ-CLK1 on cell viability, proliferation, and apoptosis

**Figure 4.**
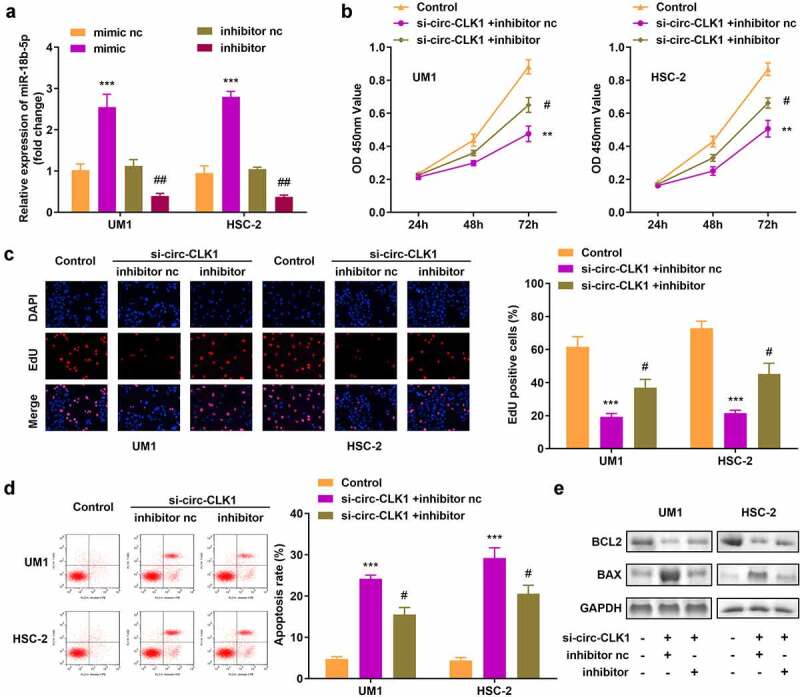
**Inhibition of miR-18b-5p reverses the effects of circular-CDC like kinase 1 (circ-CLK1) on cell viability, proliferation, and apoptosis**. (a) Expression of miR-18b-5p in UM1 and HSC-2 cells; (b) Cell viability of UM1 and HSC-2 cells; (c) Cell proliferation of UM1 and HSC-2 cells; (d) Apoptosis of UM1 and HSC-2 cells; (e) Protein expression of Bcl-2-associated X protein (BAX) and B-cell lymphoma-2 (BCL2). #*P* < 0.05, ##*P* < 0.01, **P < 0.01, ***P < 0.001 versus control.

The expression of miR-18b-5p was increased and decreased, respectively, by transfecting either miR-18b-5p mimics or inhibitors in UM1 and HSC-2 cells (Figure 4a). Inhibition of miR-18b-5p attenuated the effects of circ-CLK1 knockdown on the viability and proliferation of UM1 and HSC-2 cells (Figure 4b, 4c). Moreover, downregulation of miR-18b-5p expression also reduced apoptosis, while BCL2 and BAX expression was increased and decreased, respectively (Figure 4d, 4e).

### miR-18b-5p directly targeted YBX2

**Figure 5.**
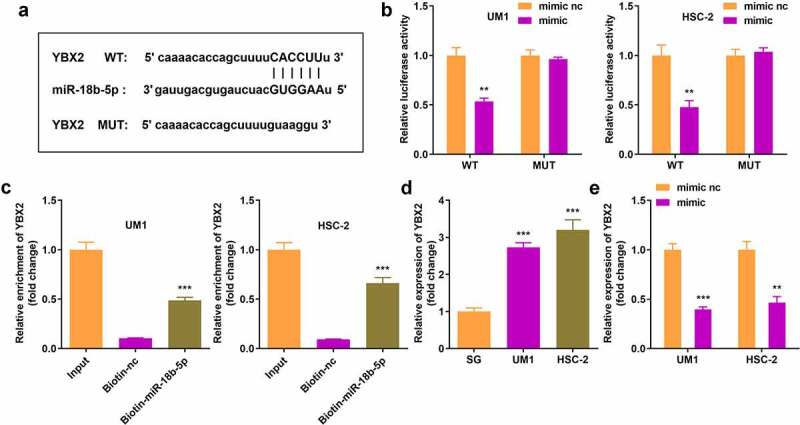
**miR-18b-5p directly targets Y-box protein 2 (YBX2)**. (a) Binding sites of YBX2 and miR-18b-5p; (b) The luciferase activity of UM1 and HSC-2 cells co-transfected with luciferase reporter vector containing YBX2 and miR-18b-5p overexpression vector; (c) YBX2 expression after pulling down with miR-18b-5p; (d) YBX2 expression in UM1 and HSC-2 cells. (e) Expression of YBX2 in cells with miR-18b-5p overexpression. **P < 0.01, ***P < 0.001 versus control.

YBX2 was a potential target gene for miR-18b-5p identified through *in silico* analyses, and was further confirmed by the dual luciferase reporter and RNA pull-down assays (Figure 5a–5c). In addition, YBX2 expression notably increased in UM1 and HSC-2 cells, but decreased in miR-18b-5p overexpressed tumor cells (Figure 5d, 5e).

### YBX2 overexpression reversed the effects of miR-18b-5p on cell viability, proliferation, and apoptosis

**Figure 6.**
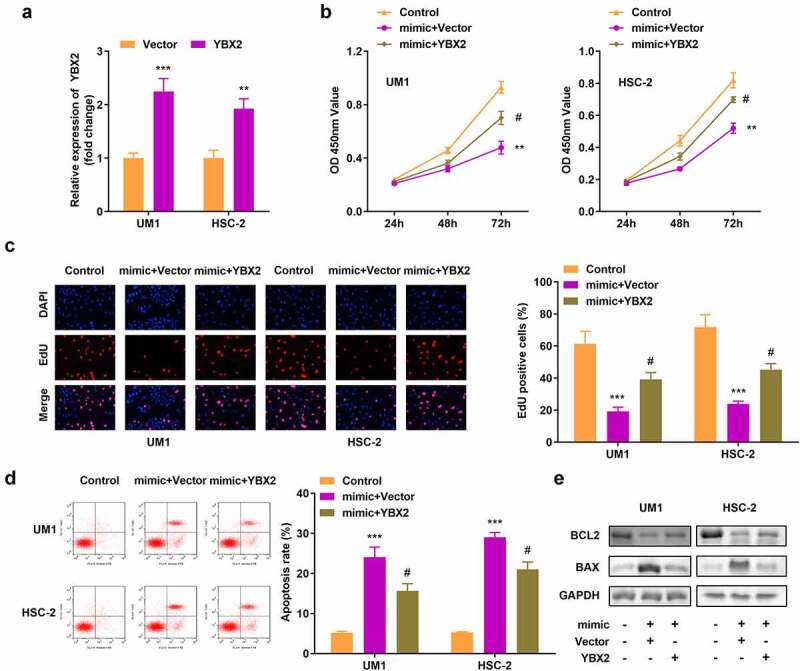
**Overexpression of Y-box protein 2 (YBX2) reverses the effects of miR-18b-5p on cell viability, proliferation, and apoptosis**. (a) YBX2 expression in UM1 and HSC-2 cells; (b) Cell viability of UM1 and HSC-2 cells; (c) Cell proliferation of UM1 and HSC-2 cells; (d) Apoptosis of UM1 and HSC-2 cells; (e) Protein expression of Bcl-2-associated X protein (BAX) and B-cell lymphoma-2 (BCL2). #*P* < 0.05, **P < 0.01, ***P < 0.001 versus control.

We transfected the YBX2 overexpression vector into UM1 and HSC-2 cells and observed an increase in its expression (Figure 6a). In addition, YBX2 overexpression reversed the decrease in tumor cell viability and proliferation resulting from the overexpression of miR-18b-5p (Figure 6b, 6c). Compared to cells with miR-18b-5p overexpression, apoptosis declined following YBX2 overexpression (Figure 6d), and BCL2 increased, while BAX decreased in cells with YBX2 overexpression (Figure 6e).

## Discussion

OSCC is the most common type of head and neck cancer [32], while Asia has the highest incidence and mortality rates worldwide [33]. It has been reported that circRNAs play a regulatory role in OSCC. For instance, circRNA_0000140 inhibits the growth and metastasis of OSCC by regulating the Hippo signaling pathway [34]. CircRNA_002178 promotes OSCC proliferation and migration by activating the Akt/mTOR signaling pathway [35]. The circ-CLK1 in this study was reported to play a vital role in hepatocellular carcinoma [36]. The expression of circ-CLK1 in our collected samples and OSCC cell lines was abnormally increased, and inhibition of circ-CLK1 *in vitro* promoted tumor cell apoptosis and reduced OSCC cell viability. This suggests that circ-CLK1 has a potential regulatory function in OSCC.

It is known that circRNAs can regulate miRNAs through ceRNA mechanisms, thereby affecting the activity of target genes [37]. Here, we predicted the target miRNA of circ-CLK1 to obtain miR-18b-5p, which has a regulatory effect on ovarian and liver cancer [38,39], but has not been reported in OSCC. In this study, miR-18b-5p was highly expressed in OSCC cells and rescued the impact of circ-CLK1 knockdown on cell viability and apoptosis, indicating that miR-18b-5p is a key factor in the circ-CLK1-mediated regulation of OSCC pathogenesis.

In the ceRNA network, circRNA protects downstream genes by binding to miRNAs [15], thereby up-regulating their expression. We predicted that YBX2, which regulates DNA transcription and translation, is a downstream gene of miR-18b-5p. YBX2 is reported to be regulated by lncRNA, which can affect the occurrence and development of OSCC [40,41]. In this study, YBX2 was highly expressed in OSCC cells, and overexpression inhibited OSCC cell apoptosis and enhanced tumor cell viability.

## Conclusion

Overall, as shown in graphical abstract, our study proves that circ-CLK1 can serve as a potential biomarker for OSCC, and the mechanism by which circ-CLK1 regulates apoptosis through the miR-18b-5p/YBX2 axis promotes the development of OSCC, which is important for molecular targeted therapy of OSCC.

## Research Highlights

1. circ-CLK1 is highly expressed in OSCC.
2. circ-CLK1 directly targets miR-18b-5p.
3. miR-18b-5p directly targets YBX2.
4. circ-CLK1 inhibited the apoptosis of OSCC cells through the miR-18b-5p/YBX2 axis.
